# Mechanisms of FH Protection Against Neovascular AMD

**DOI:** 10.3389/fimmu.2020.00443

**Published:** 2020-04-03

**Authors:** Céline Borras, Kimberley Delaunay, Yousri Slaoui, Toufik Abache, Sylvie Jorieux, Marie-Christine Naud, Mohamed El Sanharawi, Emmanuelle Gelize, Patricia Lassiaz, Na An, Laura Kowalczuk, Cédric Ayassami, Alexandre Moulin, Francine Behar-Cohen, Frédéric Mascarelli, Virginie Dinet

**Affiliations:** ^1^Centre de Recherche des Cordeliers, Inserm UMR1138, Université de Paris, Sorbonne Université, Paris, France; ^2^Université Paris Diderot, Sorbonne Paris Cité, Paris, France; ^3^INSERM, U1138, Paris, France; ^4^Université Pierre et Marie Curie - Paris6, UMRS1138, Paris, France; ^5^Laboratoire de Mathématiques et Applications UMR 7348, CNRS, Poitiers, France; ^6^Laboratoire Français du Fractionnement et des Biotechnologies (LFB), Lille, France; ^7^Department of Ophthalmology of Lausanne, University Jules Gonin Eye Hospital, Lausanne, Switzerland; ^8^Ophtalmopole, Hôpital Cochin Assistance Publique Hôpitaux de Paris, Paris, France

**Keywords:** AMD, complement factor H, FH Y402H polymorphism, TSP-1, therapeutic target

## Abstract

A common allele (402H) of the complement factor H (FH) gene is the major risk factor for age-related macular degeneration (AMD), the leading cause of blindness in the elderly population. Development and progression of AMD involves vascular and inflammatory components partly by deregulation of the alternative pathway of the complement system (AP). The loss of central vision results from atrophy and/or from abnormal neovascularization arising from the choroid. The functional link between FH, the main inhibitor of AP, and choroidal neovascularization (CNV) in AMD remains unclear. In a murine model of CNV used as a model for neovascular AMD (nAMD), intraocular human recombinant FH (recFH) reduced CNV as efficiently as currently used anti-VEGF (vascular endothelial growth factor) antibody, decreasing deposition of C3 cleavage fragments, membrane attack complex (MAC), and microglia/macrophage recruitment markers in the CNV lesion site. In sharp contrast, recFH carrying the H402 risk variant had no effect on CNV indicating a causal link to disease etiology. Only the recFH NT^al^ region (recFH1-7), containing the CCPs1-4 C3-convertase inhibition domains and the CCP7 binding domain, exerted all differential biological effects. The CT^al^ region (recFH7-20) containing the CCP7 and CCPs19-20 binding domains was antiangiogenic but did not reduce the microglia/macrophage recruitment. The antiangiogenic effect of both recFH1-20 and recFH-CCP7-20 resulted from thrombospondin-1 (TSP-1) upregulation independently of the C3 cleavage fragments generation. This study provides insight on the mechanistic role of FH in nAMD and invites to reconsider its therapeutic potential.

## Introduction

Age-related macular degeneration (AMD) is the leading cause of vision loss over the age of 55, affecting 30-50 million individuals worldwide (1). Excessive complement activation and genetic variants in the complement alternative pathway (AP) compounds [Factor H (FH), Factor I (FI), and Complement C3 (C3)] are widely accepted as a contributor to AMD. The 1277 T>C polymorphism in the *cfh* DNA sequence, leading to a substitution of tyrosine to histidine at position 402 (Y402H), is the major risk factor for atrophic and neovascular (nAMD or wet AMD) forms of the disease, increasing the risk of AMD by 5–7% in homozygotes (2). Only nAMD, in which choroidal neovascularization (CNV) causes photoreceptor death (3), is amenable to treatment using repeated intravitreous (IVT) injections of anti-VEGF (vascular endothelial growth factor) agents (4). Nevertheless, about 20-30% of AMD patients respond poorly to anti-VEGFs (5), and long-term VEGF neutralization may favor macular atrophy (6) as VEGF is trophic to the retina (7) and the choroid (8).

FH, the main soluble regulator of the AP, acts in body fluids as well as on cell surfaces by preventing the formation and accelerating the decay of the C3/C5 convertases and by assisting the degradation of C3b by FI (cofactor activity). FH is composed of 20 complement control protein (CCP) units, of which CCPs1***-***4 are responsible for both the cofactor- and decay-accelerating activity of FH (9). CCPs6-8 and CCPs19-20 carry two binding sites to glycosaminoglycans (GAGs) on host cells, and on the Bruch's membrane (10), protecting them against complement activation (11). Produced mainly by the liver, FH is also produced locally in the eye (12), particularly in the retinal pigment epithelium (RPE) and choroid (12, 13). Synthesis of FH by RPE cells is downregulated by oxidized photoreceptor outer segments (12), suggesting that oxidative products accumulating with aging may activate the AP and promote local inflammation that favors neovascularization. Moreover, aging induces a decrease in the amount of sulfated GAGs on Bruch's membrane in the human eye (14), inducing a lower capacity to bind FH, especially the risk variant, FH_402H_. How the FH polymorphism influences AMD, including the development of CNV and which part of FH protects against CNV, remains imperfectly understood. The FH_402H_ variant is suspected of having reduced protection against the membrane attack complex (MAC/C5b-9) deposition at the level of RPE/choroid complex (15), but its impact on the FH binding to the Bruch's membrane and RPE cells is controversial (10, 16). While the deficiency of downregulators of AP favors the development of experimental CNV in mice (17, 18), the local or systemic inhibition of AP has controversial effects on CNV (19, 20). At a time when strategies to limit AP activation have failed to demonstrate preventive effects in the atrophic form of AMD (21, 22), the question of whether recombinant FH can be a therapy alternative for CNV remains a subject of debate. Having demonstrated that recombinant human FH (recFH1-20) reduces CNV in a murine CNV model, a reliable model for therapy screening in nAMD (18–20, 23), we performed a molecular dissection of the FH domains to elucidate the cellular and molecular mechanisms responsible for the antiangiogenic effects. We showed that (i) the antiangiogenic effect of FH is concomitant with a decrease in the formation/deposit of MAC; (ii) the CCPs1-7 fragment mimics the functional effects of full-length FH, while CCPs7-20 fragment inhibits CNV *via* a different pathway; (iii) the effect of FH passes through an upregulation of thrombospondin-1 (TSP-1) level. Moreover, recombinant FH carrying the H402 risk variant has no protecting effect against CNV, indicating a causal link to nAMD.

## Materials and Methods

### Animals

Three-month-old male Long Evans rats, male C57Bl/6J (Janvier, France) and male *ccl2*^−/−^ mice on a C57Bl/6j background (kindly provided by EDTA Center, Orléans, France), were used in this study. All procedures conformed to the resolution on the use of animals in research of the Association for Research in Vision and Ophthalmology and to the guidelines of the Institut National de la Santé et de la Recherche Médicale Committee on Animal Research. This study was carried out in accordance with the principles of the Basel declaration and recommendations of the “Ministère de l'Education Supérieur et de la Recherche Française,” animal ethics committee N°005. The protocol was approved by the animal ethics committee N°005.

For the study, several experiments of four animals per group were used: a control group with PBS (Phosphate-Buffered Saline)-intravitreous (IVT) injection, and a group with recFH1-20 or one of its fragments (diluted in PBS) IVT injection. Before treating the eyes, the mice or rats were anesthetized by intraperitoneal injection of a mixture of ketamine (20%, cat. KET205, Virbac, France)/xylaxine (40%, cat. ROMOO1, Bayer, France)/NaCl (40%, cat.2780-291, VWR, Belgium) 40 mg/kg. Their pupils were anesthetized locally and dilated with application to the cornea of, respectively, tetracaine 1% (cat. 24095101, Thea, France) and mydriaticum solution 2 mg/0.4 ml (cat. 24080201, Thea, France). Four days postlaser photocoagulation, PBS or RecFH1-20 or one of its fragments was injected into the vitreous of rats (final volume 3 μl) or mice (final volume 1 μl) at different concentrations (0.6, 0.06, and 0.006 μM) (Table 1). To avoid pressure-induced damage in mice eye, only 1 μl was injected in the vitreous. To investigate a combination treatment of recFH (0.1 μM) and of anti-VEGF (0.1 μM, R&D system, France), both of them were IVT injected in the same time, concomitant with impacts of laser. For neutralization of thrombospondin-1 (TSP-1) studies, a specific mouse anti-TSP-1 rat antibody (Table 2, Merck, France) was injected (6 μM/3 μl), 10 min after IVT injection of recFH1-20 (0.6 μM) or of recFH7-20 solution (0.6 μM), at day 4 post-laser in the vitreous of rats submitted to laser photocoagulation. IVT coinjection of PBS/PBS, PBS/recFH (1–20 or 7–20), anti-TSP-1/recFH (1–20 or 7–20), and anti-TSP-1/PBS was repeated at least three times with four animals per experimental group in both eyes. For control experiments, mouse IgG solution (6 μM, CSB-NP00581m, Interchim, France) was IVT coinjected, in place of mouse anti-TSP-1 IgG, with recFH (1–20 or 7–20) or PBS. At day 14 postlaser photocoagulation, euthanasia was performed at the same time of the day (11:00 a.m.) by placing animals in a CO_2_ chamber, followed by cervical dislocation. The globes of the animals were collected.

**Table 1:** Concentration of recFH fragments.

**recFH Fragments (CCPs)**	**Stock solution (mg/ml)**	**Injected concentration (μM)**	**Rat-IVT volume (μl)**	**Mice-IVT volume (μl)**
1–20 (155 kDa)	2	0.6–0.006	3	1
1–18 (122 kDa)	2.7	0.6	3	–
1–7 (50 kDa)	0.266	0.6	3	–
1–6 (44 kDa)	0.868	0.6	3	–
7–20 (96 kDa)	0.814	0.6	3	1
8–20 (90 kDa)	1.1	0.6	3	–
1–20_402H_ (155 kDa)	2	0.6	3	–
1–7_402H_ (50 kDa)	0.583	0.6	3	–

**Table 2:** List of lectin and antibodies.

**Lectin/Antibody**	**Clone/species**	**Manufacturer**	**Country**	**Catalog number**
ISB4	FITC-conjugated	Vector Labs	France	FL-1201
Factor H	OX-24 (Mouse)	BIO-RAD	France	MCA509G
Factor H	Polyclonal (Goat)	Sigma	France	SAB2500260
TSP-1 (anti-thrombospondin)	A6.1 (Mouse)	Merck	France	BA24
CD68	ED1 (Rat)	AbD Serotec	France	NC9625648
VEGF	Polyclonal (Rat)	R&D Systems	France	AF564
C5b-9 (MAC)	Polyclonal (Rabbit)	Abcam	France	Ab55811
C3	bH6 (Mouse)	Abcam	France	Ab90814
C3b fragments	Monoclonal (Mouse)	CliniSciences	France	HM1065
Actin	Polyclonal (Rabbit)	ThermoFisher	France	PA5-78715

### Production, Purification, and Characterization of Human Plasma and Recombinant FH and Its Fragments

Plasma FH (plFH) was purified from pooled human plasma according to a process developed for the preparation of therapeutic product. RecFH1-20 and recFH1-20_402H_ were produced from stable pools of PER.C6 human cell line, established according to the PER.C6 Know-How File (Version 2009, Crucell, The Netherlands). Briefly, the human *cfh* cDNA sequence of reFH1-20_402H_ was obtained by gene synthesis with a codon optimization and cloned into a pCDNA2001neo expression vector using the In-Fusion HD EcoDry kit (Clontech, Mountain View, CA, USA). The DNA sequence of recFH1-20 was obtained by site-directed mutagenesis of recFH1-20_402H_ sequence. Cell transfections were performed at room temperature by electroporation of 8 μg of expression vectors for 6.10^6^ cells. Transfected cells were then cultured at 36.5 and 5% CO2 in Permab medium during 48 h before to apply a selection pressure with neomycin at 1 g/L to establish stable pools. The FH production from stable pools was performed in batch mode at 36.5 and 5% CO2 in Permab medium. After 7 days, the supernatants were recovered and clarified using disposable depth filters. Then, the recFH molecules were purified from supernatants by two ion exchange chromatography steps. Both plFH and recFH1-20 were pure >90% as determined by SDS-PAGE (Supplementary Data 1A). Structural and functional analysis showed that recFH molecules were upstanding. RecFH fragments were obtained by PCR assembly with the In Fusion cloning kit (Clontech, Mountain View, CA, USA) using the full-length *cfh* cDNA as matrix. A GDSGS or GGSG linker and a hexa-histidine tag were added at the C-terminal part of the recFH fragments. The production of the recFH fragments was carried out by transient expression into the human cell line HEK293 Freestyle (Thermofisher, Carlsbad, CA 92008 USA) using the pCEP4 vector and 293Fectin transfection reagent (Thermofisher, Carlsbad, CA 92008 USA). After 7 days of production, the supernatants were harvested, clarified by centrifugation, and purified by affinity chromatography on a Ni-NTA column by means of the hexa-histidine tag added at the C-terminal part of the recFH fragments, except the untagged fragment recFH1-18, which was purified like full-length recFH.

#### Decay Accelerating Convertase

Microtiter plate wells were coated overnight at +4°C with 250 ng of purified C3b (Calbiochem, France). Generation of C3bBb complex was achieved by addition of factor B (400 ng) and factor D (30 ng) in the presence of 1.5 mM NiCl_2_ and 2 mM NaCl into a final volume of 100 μl. After 2 h incubation at 34°C of the mixtures, serial dilutions of the recFH1-20 and fragment were added and dissociation of the complexes monitored at further time points during 30 min at +34°C. Intact complexes on the Enzyme-linked Immunosorbent Assay (ELISA) plates were detected using antihuman factor B antibody and peroxidase conjugated goat antihuman IgG (H+L) (Calbiochem, France).

#### Sheep Red Blood Cells Hemolysis

Sheep red blood cells (SRBCs) were washed 3x NaCl 0.9% and resuspended in HBS (Hepes 10 mM, NaCl 144 mM, pH 7.2)/10 mM EGTA/7 mM MgCl_2_. Serial dilutions of recFH or fragments (0, 15, 22, 30, and 45 pM) were added into the mixture of normal and FH depleted human plasma (CompTech, USA), then 4.10^6^ SRBCs were added for 30 min at 37°C. The reaction was stopped by adding HBS/2mM EDTA buffer, and the mixture was centrifuged. Two hundred microliters of supernatant were removed, and optical density (OD) read at 414 nm with a plate reader. Results were expressed as percentage of hemolysis activity as compared to plFH.

### Laser Photocoagulation

CNV was induced by an Argon laser photocoagulator (532 nm) mounted on a slit lamp. For immunofluorescence experiments, five separate photocoagulation lesions (rats: 50 μm spot size, 0.1 ms duration and 175 mW power and for mice: 50 μm spot size, 0.05 ms duration and 250 mW power) around and close to the optic nerve (1–2 disc diameters away from the papillae) were created in both eyes of each experimental animals group. The rat and mice eyes were not treated in the same manner, as the eyes of mice were smaller than those of rats. For RT-Q-PCR and Western blot experiments, 10 separate laser spots were uniformly realized on the total surface of the retina. The presence of a bubble witnessed the rupture of Bruch's membrane and confirmed a successful laser impact.

### Fluorescein Angiography

Fluorescein angiography (FA) was performed 12 days after laser induction. After pupil dilatation, fluorescein (0.2 ml of 10% fluorescein solution diluted in saline buffer, Thea, France) was injected intravenously in the tail of rats. Phase angiograms were recorded at 3–5 min after fluorescein injection. Simultaneously, infrared images (IR) were acquired to detect the site and effective presence of laser burn. Grading of vascular leakage was performed on fluorescein angiograms showing the mean angiographic score per impact in each experimental group at day 12 postlaser (five impacts per eye in each four animals per group). Angiographic scores were established by two blinded observers according to the following criteria: grade 0, no hyperfluorescence; grade 1, slight hyperfluorescence with no increase in intensity nor in size; grade 2, hyperfluorescence increasing in intensity but not in size; grade 3, hyperfluorescence increasing both in intensity and size; grade 4, hyperfluorescence size increase more than 2-diameter of the initial laser burn.

### Flat Mount Preparation and CNV Quantifications

Flat mounts of the RPE–choroid–sclera complex of Long Evans rats were prepared. The enucleated eyes were incised at the limbus and immediately fixed at 4°C for 30 min with 4% paraformaldehyde (PAF, cat. Sc-281692, Santa Cruz, USA) prepared in PBS, washed three times with PBS, before the anterior segments were dissected out. The neuro-retina was removed from the RPE/choroid/sclera complex. Five radial cuts were made from the edge of the eyecup to the equator, and the choroid/RPE/sclera complex was flat-mounted, with the sclera facing down. Postfixation with PAF4% for 15 min at 4°C was performed, and RPE–choroid–sclera complex further processed as follows: wash three times with PBS plus 1% Triton X-100 (cat. T8787, Merck, France), incubation overnight with normal goat serum (cat. Ab7481, Abcam, France) 10% diluted in PBS/Triton X-100 1% to block nonspecific sites and incubation 2 days at 4°C in Fluorescein IsoThioCyanate (FITC) labeled *Griffonia Simplicifolia* I isolectin B4 (ISB4, Vector Labs, France) diluted 1:200 in PBS-Triton1% (Table 2). After this incubation, the samples were washed thoroughly with PBS-Triton1% and flat-mounted between a slide and a coverslip using Dako gel mounting (Dako, France). Flat mounts were observed under a Zeiss confocal Imaging system (LSM710, Zeiss, France) and Z-stack images (1 μm thickness of each optical section, seven optical sections in one Z-stack) of CNV-injured area were captured with a digital video camera coupled to computer system. Bruch ruptures could be easily observed at each laser spot. Area of fluorescence signal (CNV size) was measured using ImageJ software (National Institute of Health, Bethesda, MD). The fluorescence color in the laser spot represents CNV complex. The summation of the entire fluorescent area on Z-stack images from the top to the bottom of the CNV was used as an index for the CNV volume. If the CNV was <3% of the total laser spot area, it was graded as negative while CNV >3% was considered positive.

### Immunofluorescence

For immunofluorescence experiments studies, all samples were processed in the same manner. The RPE/choroid/sclera flat mounts were incubated in PBS1X/BSA 0.1%, permeabilized in 0.3% Triton X-100 (cat. T8787, Merck, France) for 15 min, saturated with normal goat serum (cat. Ab7481, Abcam, France) 10%/PBS1X one night at 4°C, and then stained two days at 4°C in selective primary antibodies diluted in 0.3% Triton X-100 in PBS (Table 2): FITC labeled *Griffonia Simplicifolia I isolectin* B4 (FITC-ISB4, 1:200, Vector Labs, France), rabbit polyclonal anti-C5b-9/MAC (1:500, Abcam, France), mouse monolonal anti-TSP-1 (1:200, Merck, France) and rat polyclonal anti-CD68 (1:300, AbD Serotec, France) antibodies. After washing three times in PBS/triton 0.3%, flat mounting preparations were incubated in a solution of 1:200 of secondary antibody conjugated to Alexa (red 594nm, purple 647 nm or green 488 nm; Molecular Probes, France) and corresponding to the primary antibody for 60 min at room temperature. The slides were then washed three times in PBS/Triton 0.3% (10 min/RT), stained for 5-10 min with DAPI, and washed three times in PBS1X. The flat mounts or sections were then mounted with Dako solution (Dako, France) and then examined with a Zeiss confocal Imaging system (LSM710, Zeiss, France) and Z-stack images (1 μm thickness of each optical section; seven optical sections in one Z-stack) of laser-injured area were captured with a digital video camera coupled to computer system. Bruch ruptures could be easily observed at each laser spot. As a control, the primary antibody was omitted: no staining was observed in any control (Supplementary Data 3B, 5A). Identical exposure parameters were used to compare the fluorescence intensity of staining in control CNV-experimental group (PBS IVT injection) with that in recFH CNV-experimental groups (recFH or its fragment IVT injection). Mean intensity (mean gray value, within range from 0 to 255) of green or red fluorescence in area of laser injury was measured using ImageJ Software (National Institute of Health, Bethesda, MD). If the CNV was <3% of the total laser spot area, it was graded as negative while CNV >3% was considered positive. Each experiment including IVT injections was repeated three times.

### Western Blot Analysis

Total protein was extracted from retinal tissue (RPE–choroid–sclera and neural retina). The tissue was homogenized and solubilized in ice-cold PBS containing protease inhibitors (cat. Ab201119, Abcam, France) plus NP40 0.1%. For western blot analysis, we used 30 μg of extracted proteins for each point. Briefly, electrophoresis was performed by SDS-PAGE 4–12% Tris-gel and the separated proteins were transferred to nitrocellulose membrane (cat. IPVH00010-Immobilon; Merck, France). Blocking of nonspecific binding was achieved by placing the membrane in 5% no fat dry milk diluted in TBS 1X solution. Mouse monoclonal antihuman FH (Table 2, 1:3000, Bio-RAD, France), goat polyclonal anti-FH (Table 2, 1/2000, Sigma, France), mouse monoclonal anti-C3b fragments (Table 2, 1:2000, CliniSciences, France), mouse monoclonal anti-C3 (Table 2, 1:3000, Abcam, France), rabbit polyclonal antiactin (Table 2, 1:3000, Thermo Fisher, France), or mouse monoclonal anti-TSP-1 (Table 2, 1:500, Merck, France) antibodies were incubated as the primary antibodies overnight at 4°C, and then the blots were washed with TBS1X/milk 1% and incubated separately with the corresponding second antibody coupled to horseradish peroxidase (1:3000, cat. 9003-99-0, Abcam, France). Blots were developed using the enhanced chemiluminescence Western blotting detection system “ECL-Plus” (Cat. A38555, Amersham Pharmacia Biotech, Arlington Heights, IL) according to manufacturer's recommendations. Semiquantification of protein level was accomplished by analyzing the intensity of the bands using ImageJ Software (National Institute of Health, Bethesda, MD).

### Quantitative Real-Time Polymerase Chain Reaction (Q-PCR)

Retinal samples from at least three animals were pooled for each condition. Total RNA from RPE/choroid/sclera and neural retina was isolated with TRIZOL reagent (Invitrogen, France) according to the manufacturer's instructions, and Superscript II Reverse Transcriptase (Invitrogen, France) was used to reverse transcribe 1 μg of total RNA. Amplification reaction assays containing 1 × SYBR Green PCR Mastermix (Applied Biosystems, France) were realized following the company instructions. All real-time PCR oligonucleotide primers, previously experimentally validated by RT-Q-PCR, agarose gel analysis, and BLASTPrimers, were designed such that amplicon sizes ranged from 50 to 250 bps (Table 3). A hot start at 95°C for 5 min was followed by 40 cycles at 95°C for 15 s and 60°C for 1 min with the 7300 SDS thermal cycler (Applied Biosystems, France). Controls with no reverse transcriptase were run for each assay to confirm the lack of genomic DNA contamination. Control RT-Q-PCR reactions were performed without cDNA templates. Two reference genes (*actin* and *cyclophilin)* were used. The ABI Prism 7700 Sequence Detection System was used for relative quantification of gene expression. At least three different experiments were conducted for each gene and sample (*n* = 10 retinas per sample), and with each experiment, individual sample was run in triplicate and the Ct of each well was recorded at the end of the reaction. The average and standard deviation of the three Cts were calculated. Gene expression levels were normalized to *actin* or *cyclophilin* for each retinal tissue sample and calculated relative to PBS-IVT injected tissue (control) with the following equation: relative expression = 2^−(sampleΔ*Ct*−*controlΔCt*)^ where ΔCt = mean Ct(target) – mean Ct(actin).

**Table 3:** List of primers forward and reverse used for Q-PCR experiments.

	**Forward**	**Reverse**
**ANGIOGENESIS**		
*vegfA*	ACGAAAGCGCAAGAAATCCC	TTAACTCAAGCTGCCTCGCC
*flt1*	CGACACTCTTTTGGCTCCTTCTAAC	TGACAGGTAGTCCGTCTTTACTTCG
*flK1*	TCTCGTACGGACCGTTAAGC	CTCATCCAAGGGCAGTTCAT
*pedf*	AGTTACGAAGGCGAAGTCACCAAGTC	GCCCGGTGTTCCACCTGAGTC
*tsp-1*	TCGGGGCAGGAAGACTATGA	ACTGGGCAGGGTTGTAATGG
**INFLAMMATION**		
*ccl2*	GCAAGATGATCCCAATGAGT	GTCAGCACAGATCTCTCTCTT
*ccr2*	GACCGAGTGAGCTCAACATTT	AACCCAACTGAGACTTCTTGC

### Statistical Analyses

Statistical analyses were performed by computer (GraphPAD Software Inc). For western blot and Q-PCR, data are expressed as means ± SEM and were analyzed and comparison between two groups was performed using Mann–Whitney *U*-test, and differences were considered statistically significant with ^*^P < 0.05; ^**^P < 0.01; ^***^P < 0.005. In rat and mouse CNV models, in order to take into account simultaneously the correlation between the two eyes of an animal and the correlation for repeated measurements in the same eye (in case of repeated impacts), a linear mixed model for repeated measures LMMRM (known to be robust to the normality assumption) was used, including treatment (or mutation) as fixed effects and animal as a random effect.

## Results

### recFH1-20 Is as Potent as Anti-VEGF on a Murine Model of Laser-Induced CNV

To substantiate the integrity of human plFH and recFH, *in vitro* functional analysis was performed. The ability to accelerate the decay of AP C3 convertase (C3bBb), mediated by the CCPs1-4 domains of FH, was similar for plFH and recFH1-20 (Supplementary Data 1B). The regulator activity of FH on cell surfaces was tested in an FH-dependent hemolytic assay using sheep erythrocytes: plFH and recFH1-20 showed a similar protection against lysis of erythrocytes indicating the functional integrity of CCPs19-20 binding domains. Indeed, to protect erythrocyte cells from lysis, recFH must have an intact CCPs19-20 domain coupled to its CCPs1-4 C3-convertase regulated domain.

**Figure 1:**
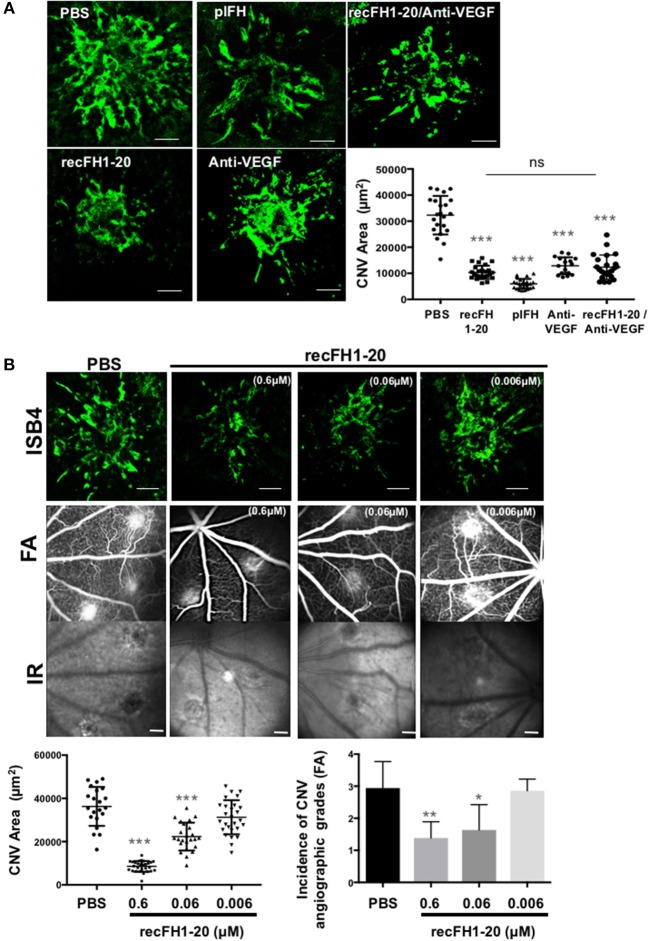
FH exhibits the same antiangiogenic activity as anti-VEGF in a CNV murine model. **(A)** ISB4 (FITC-isolectin B4, green) staining of RPE/choroid/sclera flat mounts of CNV rat model treated concomitant with IVT injection of PBS, plFH (0.6 μM), recFH1-20 (0.6 μM), anti-VEGF (0.6 μM), or with co-IVT injection of anti-VEGF and recFH1-20 (0.1 μM, respectively). Results were observed and analyzed on day 14 postlaser. ISB4 positive CNV areas (μm^2^) were expressed as mean ± SEM of the average CNV size per rat. Linear mixed model was used for statistical analyses. *p < 0.05, **p < 0.01, and ***p < 0.001. *N* = 4 animals per experimental group with impacts/eye and experiments were performed three times. Scale Bar: 100 μm. **(B)** Analysis of recFH1-20 dose-dependent antiangiogenic effect in CNV rat model. Three concentrations of recFH1-20 were used (0.6, 0.06, and 0.006 μM) for intravitreous injection (3 μl) at day 4 postlaser (D4). The CNV area labeled (green) with ISB4 was semiquantified at D14 postlaser. ISB4 positive CNV areas (μm^2^) were expressed as mean ± SEM of the average CNV size per rat. Linear mixed model was used for statistical analyses. *p < 0.05, **p < 0.01, and ***p < 0.001. Five impacts per eye in each four animals experimental group were done. Experiments were performed three times. The choroidal neovascularization leakage was analyzed by fluorescein angiography (FA). Infrared images (IR) were used to localize efficiency of laser-induced burns. Grading of vascular leakage was performed on fluorescein angiograms showing the mean angiographic score per impact experiment group at 12 days after laser. Five impacts per eye (10 per animal) were realized in four animals experimental group. Linear mixed model was used for statistical analyses. *p < 0.05 and **p < 0.01. Scale Bar: 100 μm.

Prior to efficacy evaluation, the fate of exogenous human FH was evaluated after intravitreous injection (IVT) in the native rat eye. One hour after a single IVT of plFH or recFH1-20, FH reached the RPE/choroid complex where it was detected for at least 3 days (Supplementary Data 2A). We observed no cross reaction between human recFH and rat FH antibodies in rat CNV model (Supplementary Data 2C,D, 3A). In the CNV model, laser argon is used to break Bruch's membrane, which separated RPE from choroid, and induces neovascularization from the choroid for a period of ~2 weeks. The IVT injection of plFH or recFH1-20, concomitant to laser, reduced CNV by 69% (vs. PBS, *p* < 0.001) and 76% (vs. PBS, *p* < 0.001) respectively, with an efficacy comparable to a similar molar concentration of rat blocking anti-VEGF antibody (70% vs. PBS, *p* < 0.001) (Figure 1A). IVT coinjection of recFH1-20 and anti-VEGF induced a reduction of the CNV area similar to treatment with recFH1-20 or anti-VEGF alone (Figure 1A). Since nAMD patients are treated after CNV has developed, we tested the curative effect of recFH1-20 injected at day 4 after laser injury to compensate for the decrease in endogenous FH (rat-FH) at this time (Supplementary Data 2C). Injected (D4 postlaser) recFH1-20 was observed in RPE/choroid/sclera complex at day 7 postlaser and slowly decreased until day 14 after laser (Supplementary Data 2D), suggesting a compensation of endogenous rat-FH production. RecFH1-20 reduced CNV by 76% (vs. PBS, *p* < 0.001), 35% (vs. PBS, *p* < 0.001), and 7% (vs. PBS, *p* > 0.1) at doses of 0.6, 0.06, and 0.006 μM, respectively (Figure 1B). The permeability of CNV was assessed *in vivo* by fluorescein angiography (FA) at day 12, i.e., two days before sacrifice and *ex vivo* CNV staining with FITC-isolectin B4. The antiangiogenic effect of recFH1-20 (0.6 μM vs. PBS, *P* < 0.01 and 0.06 μM vs. PBS, *P* < 0.05) was confirmed by reducing the choroidal neovascular leakage on FA compared to the PBS treatment (Figure 1B). To remain closer to human therapeutic conditions, in all furthers experiments, a single IVT injection of recFH1-20 or recFH fragments (0.6 μM) was administered on day 4 after laser induction.

### Intraocular recFH1-20 Protects From Local AP Activation and Reduces Microglia/Macrophage Recruitment

**Figure 2:**
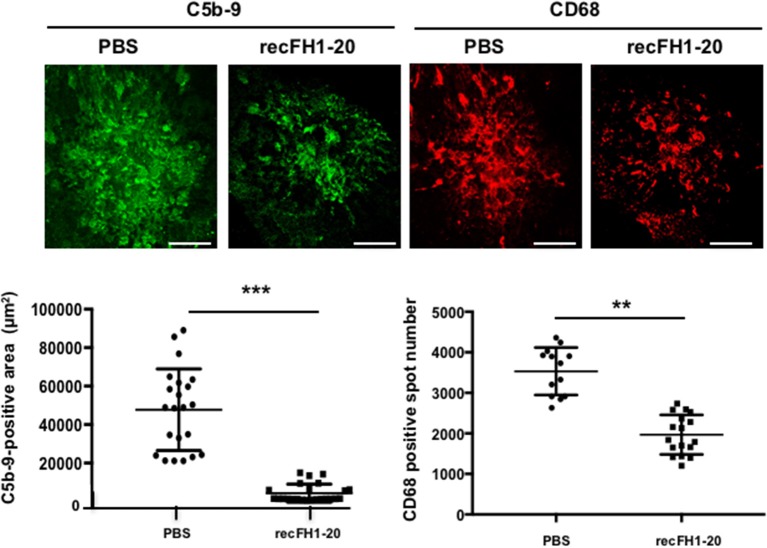
FH reduces both MAC deposit and microglia/macrophage cell recruitment. RPE/choroid/sclera flat mounts rats were immune-stained with specific markers of MAC (anti-C5b-9, green) or microglia/macrophage recruitment (anti-CD68, red) 14 days after laser. The recFH1-20 (0.6 μM) IVT injected after 4 days postlaser significantly reduced MAC deposit and microglia/macrophage recruitment as compared to the PBS-treated rat. C5b-9 positive area (μm^2^) and CD68 positive spot number were measured and were expressed as mean ± SEM per rat. Linear mixed model was used for statistical analyses. **p < 0.01, ***p < 0.001. Five impacts per eye in each four animals per experimental group were used, and experiments were performed three times. Scale Bar: 100 μm.

To understand the role per which recFH1-20 could prevent angiogenesis process, we first investigated MAC inhibition deposition. Previously, it has been shown that MAC formation primarily due to overactivation of the AP is essential for the development of laser-induced CNV (24), suggesting that an inhibition defect of this pathway could induce nAMD. Accumulation of C3 shown in the retina/choroid complex of *cfh*^−/−^ mice (25) and ocular knock-down of *cfh* increased the deposition of MAC in the RPE/choroid/sclera, together with earlier and exacerbated angiogenic response to laser induction (18), suggesting a real link between AP activation and CNV. In control PBS-injected rats, intense MAC deposit in laser CNV spot confirmed the local activation of AP (Figure 2). Together with the inhibition of CNV, postlaser injection of recFH1-20 reduced MAC staining by 85% (vs. PBS, *p* < 0.001) and also reduced recruitment of microglia/macrophages (anti-CD68 positive cells) by 37% (vs. PBS, *p* < 0.01) in the laser burn area (Figure 2). Altogether, these data demonstrated that recFH1-20 antiangiogenic activity was coupled to not only a local prevention of AP activation but also a decrease of microglia/macrophage recruitment in the CNV rat model.

### recFH1-7 Carries All the Effects of recFH1-20

**Figure 3:**
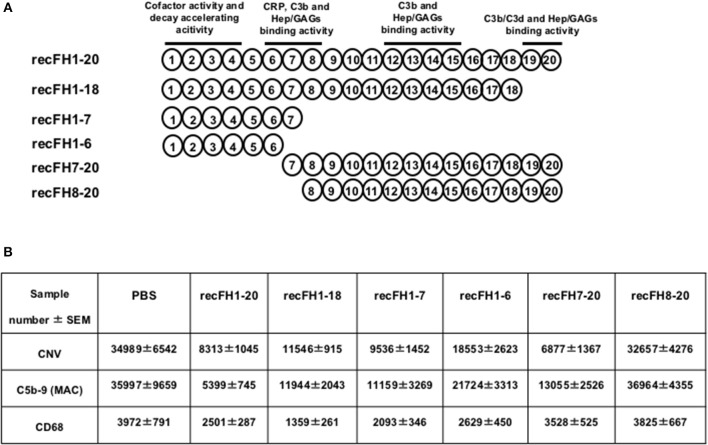
The FH antiangiogenic activity is not dependent on its C3 convertase inhibition function. **(A)** Representation of the FH domains and their functions. All recFH fragments used for CNV-rat intravitreous injection (0.6 μM) are listed. **(B)** Analysis (14 days postlaser) of IVT injected (4 days postlaser) recFH fragment effect on CNV area (ISB4), MAC production (C5b-9), and microglia/macrophage recruitment (CD68). ISB4 positive CNV area (μm^2^), C5b-9 positive area (μm^2^), and CD68 positive spot number were measured and were expressed as mean ± SEM per rat. Results were resumed in the table. Linear mixed model was used for statistical analyses. Five impacts per eye in each four animals per experimental group. Experiments were performed three times. No significance was considered with variation of level <12%.

To determine which functional domains of FH carry the antiangiogenic activity, we tested different recFH fragments on rat CNV (Figure 3A). Three CCP domains are required for the FH functions: CCPs6-8 and CCPs19-20 that contain GAGs and C3b/GAGs binding sites, and CCPs1-4 responsible for the dissociation of C3/C5 convertase. To investigate the impact of the CCPs19-20 domains, we assessed recFH1-18 or recFH1-7 that contained only one GAG-binding site (CCPs6-7) associated to the C3 convertase regulated domain (CCPs1-4). Both of them maintained *in vitro* a C3-convertase inhibition activity but did not protect erythrocytes of sheep from hemolysis, due to the deprivation of the CCPs19-20 domains (Supplementary Data 1B). RecFH1-18 and recFH1-7 prevented CNV formation by 67 and 73%, respectively (vs. PBS, *p* < 0.001) and reduced MAC formation by 67 and 69% (vs. PBS, *p* < 0.001) (Figure 3B). These data demonstrated that the CCPs8-20 and CCPs19-20 domains were not essential for the antiangiogenic and for MAC inhibition in CNV model. Furthermore, both recFH1-18 and recFH1-7 reduced microglia/macrophage recruitment at the CNV lesion by 66 and 47%, respectively (vs. PBS, *p* < 0.001) (Figure 3B). RecFH1-4 showed no effect on CNV area, recruitment of microglia/macrophages, and MAC formation (data not shown). The requirement of both CCP6 and CCP7 domains was investigated using recFH1-6 fragment. As compared to recFH1-7, recFH1-6 showed intermediate effects characterized by a decrease in CNV and MAC deposition, and in recruitment of microglia/macrophages by 47, 40, and 34% (vs. PBS, *p* < 0.001), respectively (Figure 3B), suggesting the important role of CCP7 domain in optimizing the effects of FH. Overall, these data clearly indicated that to be functional on CNV lesion, recFH must have at least the CCPs1-4 associated to CCPs6-7 binding domains.

### recFH7-20 Exerts Antiangiogenic Effects but Does Not Reduce Microglia/Macrophage Recruitment in CNV Model

**Figure 4:**
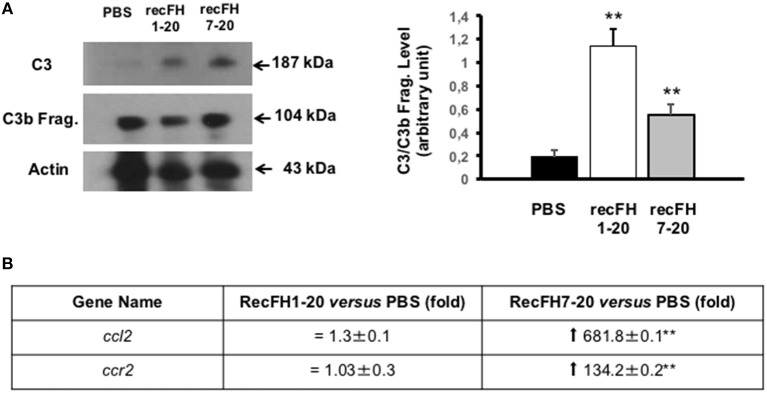
Microglia/macrophage cell recruitment depends on FH C3 convertase inhibition activity. **(A)** Western blot analysis (14 days postlaser) of C3 and its C3b cleavage products (C3b Frag.) levels in rat CNV lesion after PBS or recFH (1–20 or 7–20) intravitreous injection (0.6 μM) (day 4 postlaser). Data were expressed as means ± SEM and were analyzed and compared using Mann–Whitney *U*-test, and differences were considered statistically significant with **P < 0.01. Ten impacts per eye in each four animals per experimental group were used. Experiments were performed three times. **(B)** On Q-PCR experiments, only recFH7-20 IVT injected fragment (0.6 μM) induced an increase of *ccl2/ccr2* genes expression in the rat RPE/choroid/sclera complex at day 7 after laser induction compared to PBS injected rat. Data were expressed as means ± SEM and were analyzed and compared using Mann–Whitney *U*-test, and differences were considered statistically significant with **P < 0.01. Ten impacts per eye in each four animals per experimental group were used. Experiments were performed three times.

As expected, recFH8-20 showed no effect on CNV, on complement activation, and on microglia/macrophage recruitment (Figure 3B). We then tested recFH7-20, the C-terminal fragment encompassing the CCP7 characterized by no significant *in vitro* decay and no protective activity from sheep erythrocytes hemolysis (Supplementary Data 1B). Despite the capacity to bind membrane cells with its CCPs19-20 domains, the CCPs1-4 domains deletion in recFH7-20 fragment required ~100-fold more concentration of recFH7-20 compared to recFH to obtain the same C3 convertase inhibition function. Unexpectedly, recFH7-20 reduced CNV development and C5b-9 formation by 80 and 63%, respectively (vs. PBS, *P* < 0.001), but without effect on microglia/macrophage recruitment (Figure 3B). The absence of antiangiogenic activity of recFH8-20 compared to recFH7-20 did not result from a different biodisponibility within the retina, since recFH8-20 rapidly reached and remained in the RPE/choroid complex after IVT injection (Supplementary Data 2B). These data suggest that to reduce C5b-9 formation, the CCP7 and CCPs19-20 domains are required and are sufficient, perhaps for host cell surface binding and to reduce the formation of C3/C5 convertase complexes by competition for C3b binding. For AP-regulation activity, recFH1-20 reduced C3 cleavage to C3b fragments compared to PBS CNV-rat treatment (Figure 4A), certainly by its CCPs1-4 domains C3 convertase inhibition function. The deletion of CCPs1-4 domains observed in recFH7-20 fragment showed less inhibition C3 cleavage to C3b/bi products compared to IVT-recFH injection (Figure 4A), consistent with no effect on microglia/macrophages cells recruitment (Figure 3B). Furthermore, at day 7 postlaser, in contrast to recFH1-20, with IVT-injection of recFH7-20, both genes involved in microglia/macrophage recruitment *mcp-1* (monocyte chemoattractant protein 1)*/ccl2* and its receptor *ccr2* were significantly upregulated in the RPE/choroid complex after laser induction (Figure 4B). We demonstrated that despite its C3 convertase inhibition activity CCPs1-4 domains deletion, recFH7-20 had an antiangiogenic activity coupled with an MAC deposit reduced. The observed recFH7-20 promoted of C3 cleavage to C3b/bi products compared to recFH full length could be correlated with an increase of microglia/macrophage recruitment associated with an upregulation of *ccl2*/*ccr2* gene expression. As both recFH1-20 and recFH7-20 had an antiangiogenic activity but had opposite effect on the microglial/macrophage recruitment on the CNV lesion, we hypothesized an effect of FH on microglia/macrophages function in addition to an effect on their recruitment.

### FH Antiangiogenic Effect Is Restricted Not Only to Inhibition of Monocytes Recruitment but Also to Regulation of TSP-1 Production

**Figure 5:**
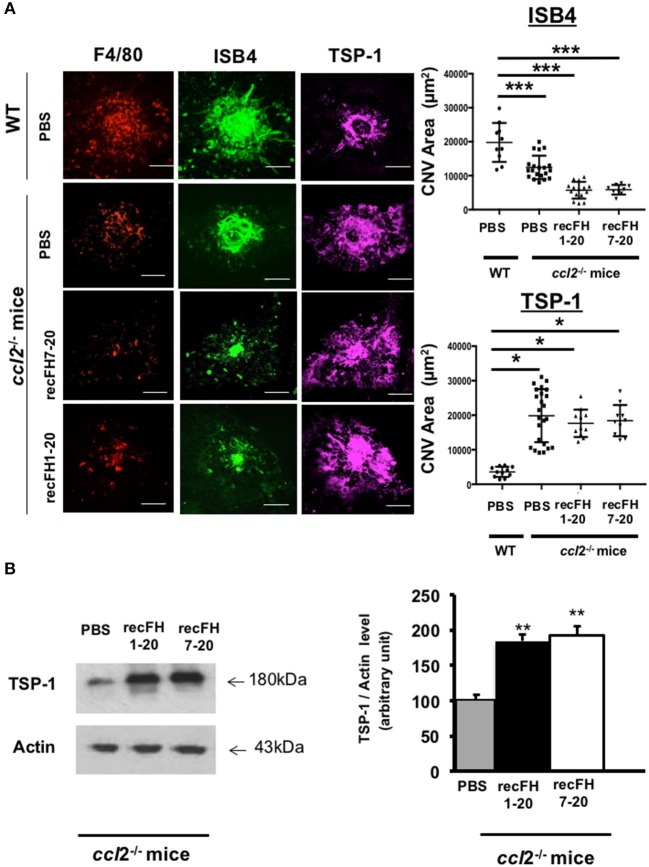
FH increases TSP-1 level previously inhibited by microglia/macrophage cells recruitment **(A)** F4/80 (red), FITC-isolectin B4 (ISB4, green), and TSP-1 (purple) staining (day 14 postlaser) on CNV- *ccl2*^−/−^ mice lesion after recFH (1–20 or 7–20) IVT injection (0.6 μM) at day 4 postlaser. Inhibition of microglia/macrophage cells recruitment reduced TSP-1 production. Five impacts per eye in each four animals per experimental group were used. Experiments were performed three times. CNV area was expressed as mean ± SEM of average CNV size per mouse. Linear mixed model was used for statistical analyses. *p < 0.05 and ***p < 0.001. Scale Bar: 100 μm. **(B)** Western blot analysis at day 14 postlaser of TSP-1 level induced by IVT injection (day 4 postlaser) of recFH1-20 (0.6 μM) or recFH7-20 (0.6 μM) in *ccl2*^−/−^ mice CNV model. Both recFH1-20 and recFH7-20 increased the TSP-1 production compared to PBS treatment. Ten impacts per eye in each four animals per experimental group were used. Experiments were performed three times. Data were expressed as means ± SEM and were analyzed and compared using Mann–Whitney *U*-test, and differences were considered statistically significant with **P < 0.01.

To evaluate whether the antiangiogenic activity of FH full length was restricted to its effect on monocytes, we used *ccl2*-knockout (*ccl2*^−/−^) mice that have reduced microglia/macrophage recruitment at the site of laser-induced CNV (Figure 5A). As expected, CNV area was reduced by 49% in *ccl2*^−/−^ mice compared to WT mice (Figure 5A), consistent with the proangiogenic effect of CCL2 (26). However, the injection of recFH1-20 or recFH7-20 induced a further CNV decrease of ~70% (vs. WT, *P* < 0.01) in *ccl2*^−/−^ mice (Figure 5A), suggesting an additive effect of FH and microglia/macrophage recruitment on antiangiogenic process.

**Figure 6:**
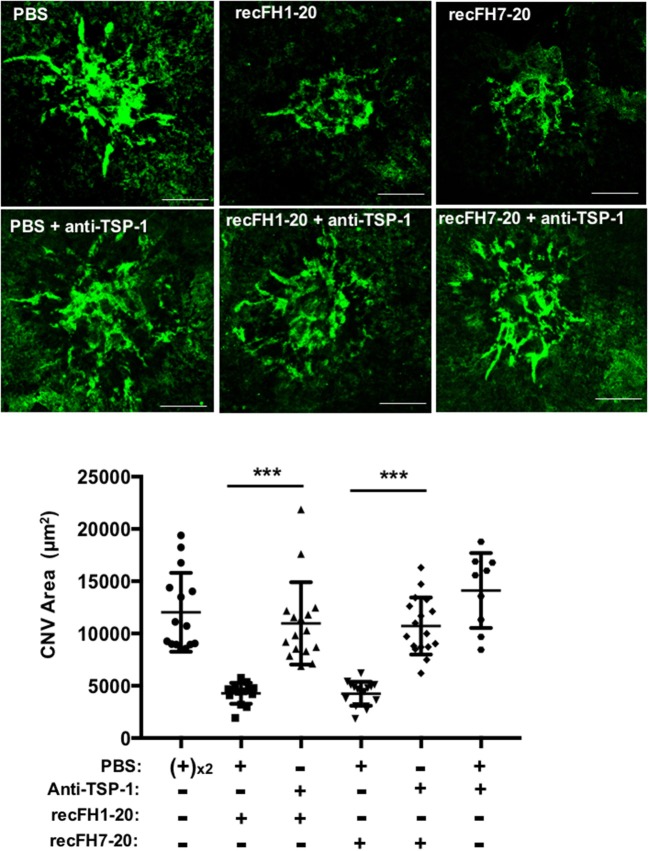
FH exerts its antiangiogenic activity at least for one part through TSP-1 function. FITC-isolectin B4 staining (ISB4, green) on RPE/choroid/sclera flat mounting laser spot was realized at day 14 postlaser for each experimental group. For treatments at day 4 postlaser, each four animals per experimental group received per eye intravitreous coinjection of either recFH1-20 (0.6 μM) or recFH7-20 (0.6 μM) and 10 min later of PBS, or in place of PBS, coinjection of mouse anti-TSP-1 antibody (6 μM). For control experimental groups, coinjection of PBS following 10 min later by injection of PBS or anti-TSP-1 (6 μM) was done. Five impacts per eye in each four animals experimental group were realized. Experiments were performed three times. ISB4-stained CNV areas were expressed as mean ± SEM of average CNV size per animal. Linear mixed model was used for statistical analyses ***p < 0.001. Scale Bar: 100 μm.

To better understand the FH antiangiogenic effect uncorrelated to microglia/macrophage recruitment observed with recFH7-20 IVT, we investigated the implicated mechanisms. As described in a recent study, the binding of FH to complement receptor 3 (CR3; CD11b/CD18) obstructs the homeostatic elimination of mononuclear phagocyte cells from the subretinal space mediated by the antiangiogenic TSP-1 binding to the CD47 receptor (27), which demonstrated a link between FH, TSP-1, and the regulation of inflammatory cells recruitment. TSP-1 immunostaining surface at the CNV site was increased in *ccl2*^−/−^ PBS-mice compared to WT PBS-injected mice (Figure 5A), which demonstrated an inhibitory effect of microglia/macrophage recruitment on TSP-1 production. The production of TSP-1 was not only regulated by inflammatory cells recruitment because IVT recFH1-20 or recFH7-20 injection in *ccl2*^−/−^ CNV-mice increased TSP-1 level in the EPR/choroid complex by 1.84 fold and 1.92 fold, respectively (vs. PBS, *p* < 0.001) compared to PBS treated CNV *ccl2*^−/−^ mice (Figure 5B), suggesting that FH was also implicated in the regulation of TSP-1 production in CNV model. IVT injection of recFH (1–20 or 7–20) also upregulated *tsp-1* gene expression in rat CNV model (Supplementary Data 4). We thus hypothesized that FH could act synergistically with *ccl2* depletion for reducing CNV process, at least in part, through regulating TSP-1 production. To determine the role of TSP-1 in the antiangiogenic effect of FH in the rat CNV model, we coinjected a blocking anti-TSP-1 antibody 10 min after the IVT recFH (recFH1-20 or recFH7-20) injection. As compared to IVT recFH (full length or recFH7-20) alone, the presence of anti-TSP-1 antibodies abolished the antiangiogenic activity of both recFH (Figure 6), providing evidence of a role of TSP-1 in the antiangiogenic activity of each recFH forms. No effect of IVT coinjection of mouse IgG and PBS or recFH (1–20 or 7–20) was detected on CNV area (Supplementary Data 5B). Altogether, these data clearly demonstrated that the FH antiangiogenic activity arose from its CCP7 binding domain coupled not only to its CCPs 1-4 domains (AP-regulation) but also to its CCPs19-20 domains (binding site), with a correlation to an upregulation of TSP-1 for both.

### FH-CCP7 Domain Is Necessary to Reduce Angiogenesis and Inflammatory Cells Recruitment Process

**Figure 7:**
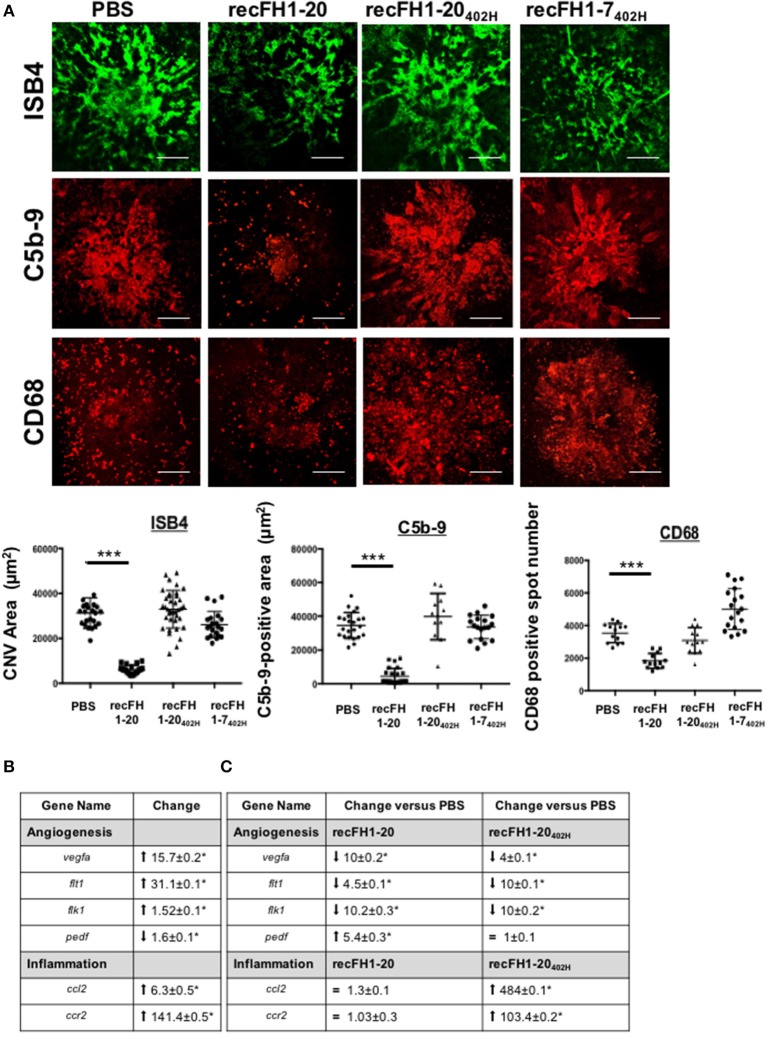
The FH-CCP7 domain is crucial for its anti-CNV process. **(A)** FITC-isolectin B4 (ISB4, green), C5b-9 (red), and CD68 (red) immunostaining analysis on RPE/choroid/sclera flat mounting laser spot at day 14 postlaser after intravitreous injection of PBS, recFH1-20 (0.6 μM) or recFH1-20_402H_ (0.6 μM) or 1-7_402H_ (0.6 μM) at day 4 postlaser. CNV area immunostainings were expressed as mean ± SEM of average CNV size per rat. Five impacts per eye in each four animals experimental group were realized. Experiments were performed three times. Linear mixed model was used for statistical analyses. ***p < 0.001. Scale Bar: 100μm. **(B,C)** Analysis by Q-PCR of recFH-CCP7 role on regulation of angiogenesis and inflammation gene expressions in CNV-rat model. Results were analyzed at day 7 postlaser **(B)** in native CNV rats or **(C)** after IVT recFH fragments (recFH1-20 or recFH1-20_402H_, 0.6 μM) injection in each eye of four animals experimental group. For each experimental group, 10 impacts were realized and experiments were performed three times. Data were analyzed and compared using Mann–Whitney *U*-test. *p*-values of 0.05 or less were considered significant.

The effect of FH_402H_ polymorphism on FH binding to GAGs, Bruch's membrane, and RPE cells is controversial (10, 16). RecFH1-20_402H_ retains its *in vitro* C3 convertase inhibition activity and its antihemolytic activity on sheep erythrocytes, due to intact CCPs1-4 and CCPs19-20 domains (Supplementary Data 1B). At the CNV lesion, recFH1-20_402H_ did not reduce CNV ISB4-staining, microglia/macrophage recruitment, and MAC formation (Figure 7A), demonstrating the importance of CCP7 module in these AMD processes. Similar results were obtained with a preventive treatment using the recFH1-20_402H_ (data not shown). The biodisponibility of recFH1-20_402H_ was similar to that of recFH1-20 after IVT, remaining in the RPE/choroid complex for at least 3 days (Supplementary Data 2B). Unlike to recFH1-7, recFH1-7_402H_ fragment did not exert any antiangiogenic or anti-inflammatory cell recruitment activity (Figure 7A), confirming that CCP 7 is determinant for angiogenic activity and inflammatory cell recruitment.

CNV is associated with transcriptomic upregulation in genes involved in inflammation recruitment and angiogenesis in the RPE/choroid CNV complex (Figure 7B). We compared the transcriptomic signatures of recFH1-20 or recFH1-20_402H_ to PBS treatment CNV rat model. We found that recFH1-20 downregulated the expression of proangiogenic gene [*vegfa* and its *receptors R1 (flt1) and R2 (flk1)*] but upregulated antiangiogenic *pedf* (Figure 7C), suggesting an imbalance in gene expression for pro- and antiangiogenic factors in favor of antiangiogenic process in the presence of FH. The FH_402H_ polymorphism also downregulated proangiogenic gene expression, but abolished the FH effect on *pedf* gene expression (Figure 7C), demonstrating a crucial role of CCP7 domain on regulation of angiogenesis gene expression. We also found that only recFH1-20_402H_ significantly upregulated the expression of *ccl2* and *ccr2* (Figure 7C), consistent with a microglia/macrophage recruitment. These data provided a link between the FH_402H_ polymorphism and nAMD.

## Discussion

The associations of gene coding proteins of the complement AP with the risk of AMD have raised the hypothesis that chronic overactivation of AP in retina/choroid plays a key role in AMD pathogenesis. But how FH, a physiological regulator of AP activation, regulates CNV remained unclear. In the present study, recFH1-20 had a strong curative and preventive antiangiogenic effect on CNV when injected locally into the eye, demonstrating that FH plays a key role in rodent CNV models (mice and rat), a clinically relevant model for testing antiangiogenic compounds for nAMD. The antiangiogenic activity of recFH1-20 was associated with a local reduction in complement activation and microglia/macrophage recruitment on CNV sites, combined with an upregulation of TSP-1 production. This is consistent with the role of inflammatory cells in experimental CNV (26) and in exudative AMD (28). AP-dependent inflammation (17, 18, 23) and MAC deposition play an important role in angiogenesis by stimulating VEGF expression in the laser-induced CNV model; moreover, inhibition of VEGF decreases local secretion of FH and other complement regulators in the eye (29), suggesting that current anti-VEGF treatments may promote local activation of AP. Treatment with recFH1-20 induced upregulation of the antiangiogenic factors *pedf* gene and the TSP-1 protein, favoring the switch from a proangiogenic to an antiangiogenic environment.

Our data showed that full-length recFH and recFH fragments containing CCPs1-4 coupled to CCP7 domain decreased CNV formation associated with reduction of MAC deposit and CD68 cells recruitment. This part of study demonstrated that to be potent on various CNV mechanisms, FH must have at least its CCP7 binding site with CCPs1-4 C3 convertase regulated domains. Unexpectedly, the recCHF7-20 fragment had the same potency in reducing CNV area and MAC deposition and differed only in its inability to reduce microglia/macrophage recruitment at the CNV injury site. Thus, the CCPs1-4 domains, which are responsible of the accelerating decay of C3/C5 convertase complexes and inactivation of C3b (iC3b), have been shown here to be mandatory to regulate the microglia/macrophage recruitment. Cell-based inflammatory responses within the RPE/choroid complex are a core feature of exudative AMD (30). Recently, a synergistic risk for AMD was found between genotype of *ccl2* (*ccl2*−2518) and C3 (31), linking in human the recruitment of microglia/macrophage cells and the complement pathway activation. Indeed, microglia/macrophage cells play a key role in local regulation of complement in the retina (32), producing VEGF (33) and enhanced laser-induced CNV (28). In a *ccl2*^−/−^ mice, in which microglia/macrophage recruitment and CNV are reduced after laser injury, we showed that low angiogenic response was associated with high TSP-1 levels. Treatment with recFH1-20 or recFH7-20 fragment further increased TSP-1, a potent antiangiogenic protein likely secreted by RPE cells (34). We found that both recFH1-20 and recFH7-20 have an antiangiogenic role despite an opposite effect on cell recruitment associated with or without the presence of CCPs1-4 domains, but all of them increased the TSP-1 production. Our hypothesis is that recFH7-20 through its CCPs7-8 and CCPs19-20 domains could bind on the microglia/macrophages or on RPE membrane cells and then would increase the level of TSP-1 release, leading to its antiangiogenic activity.

The FH is an adhesive glycoprotein with several binding sites: two sites for GAGs, CCPs6-8 and CCPs19-20, and two for C3b, CCPs1-4, and CCPs19-20. If the two C-terminal CCPs of FH are not mandatory to confer antiangiogenic activity, the binding site located on CCPs6-8, and more precisely the CCP7, is important to prevent the development of CNV consistent with the failure achieved with the recFH1-20_402H_ and the recFH8-20 fragment. It emerges from these studies that to inhibit CNV in association with reduction of MAC deposit, the FH or FH fragments must have two binding sites, one on C3b/C3dg and the other on GAGs. Since FH is a linear, flexible glycoprotein, it can be assumed that by binding with two anchor points, the FH or fragments can mask some reactive sites, particularly on C3b, to inhibit the formation of C3/C5 convertase and later, MAC. Another mechanism could be a regulation of microglia/macrophage function by FH CCP binding domain. These data consistent with structural and binding studies suggested the need for a cooperative bivalent binding of FH at the cell/membrane surface (35).

The FH-GAGs interaction forms the basis for the protection of the native GAG-coated host cell and membrane surfaces against MAC (35). However, the molecular mechanisms by which FH_402H_ participates to AMD progress are still not clear. One paradigm is proposed in which ≪zip codes≫, formed by specific protein patterns of the Bruch's membrane (GAGs, heparin, and sulfation pattern), recruit FH with higher affinity than FH_402H_, suggesting a lower level of this variant on the membrane in AMD (14). Furthermore, Toomey et al. hypothesize that FH and RPE lipoproteins (with or without oxidative modifications) compete for binding to the GAGs ≪zip codes≫ observed on the Bruch's membrane leading to increased lipoprotein accumulation and drusen formation with the presence of FH_402H_ (36). Moreover, mixed data have been observed regarding the altered affinity of the FH_402H_ variant on RPE cells (10, 16) or on a choroidal endothelial cell line (37). It remains undetermined whether the FH_402H_ polymorphism directly affects choriocapillaris and how it participates to pathogenesis of AMD, including CNV. We found that recFH1-20_402H_ did not protect against laser-induced CNV, in either curative or preventive treatment compared with the native FH, although it possesses in fluid phase a full C3 convertase inhibition activity when compared with recFH1-20 demonstrated by *in vitro* experiments. On membrane cell, recFH1-20_402H_, opposite to recFH1-20, was unable to reduce MAC deposition on the sites of the CNV lesions. Together, these findings suggest that, despite an intact C3 convertase inhibition activity, FH must bind to the membrane cells to reduce both CNV process and MAC formation, and that the CCP7 domain plays a crucial role in FH anti-CNV activities. In accordance with the bivalence and cooperativity mechanistic model of FH and because both recFH1-20_402H_ and recFH1-7_402H_ lack anticomplement activation, anti-inflammation and anti-CNV activities in the CNV model, we may speculate that FH_402H_ polymorphism alters its capacity to bind to GAG sites of the microglia/macrophages and RPE cell membranes, altering the pro-/antiangiogenic gene expression balance.

In conclusion, our data demonstrated that FH antiangiogenic activity is associated with downregulation of AP mediated by the dissociation of C3/C5 convertase (MAC formation) and/or inactivation of C3b (presence of CCPs1-4 domains) or by simply masking some active part of microglia/macrophages. The truncated form of FH, FH-like protein 1 (FHL-1) corresponding in its major structure to the recFH 1-7 fragment in our study, is the main regulatory protein in the Bruch's membrane, which is the major site of AMD pathogenesis (38, 39). While FHL-1 can passively diffuse through Bruch's membrane and is observed in drusen, FH full length cannot diffuse and coats only the periphery of the lesions (38). However, as we have shown in this study, despite the deletion of CCPs 8-20 domain, recFH1-7 fragment considerably reduced the CNV events (angiogenic and microglial/macrophage recruitment process) as effectively as the full length recFH. All together, these results clearly identify a novel mechanism of recFH1-7 fragment in AMD process (angiogenic and microglial/macrophage recruitment), which demonstrates the potential of local recFH1-7 as a therapeutic option.

The exact place of such a therapeutic compound in the landscape of anti-VEGF therapies remains to be determined by further clinical studies. In our experiments, no synergic effect was observed with coadministration of rat anti-VEGF with FH, but because FH also exerts antioxidant and anti-inflammatory action, it could have a long-term beneficial effect in a disease where neovascular processes are only part of a more complex degenerative and inflammatory process.

## Data Availability Statement

The raw data supporting the conclusions of this article will be made available by the authors, without undue reservation, to any qualified researcher.

## Ethics Statement

All procedures conformed to the resolution on the use of animals in research of the Association for Research in Vision and Ophthalmology and to the guidelines of the Institut National de la Santé et de la Recherche Médicale Committee on Animal Research. This study was carried out in accordance with the principles of the Basel declaration and recommendations of the “Ministère de l'Education Supérieur et de la Recherche Française,” animal ethics committee N°005. The protocol was approved by the animal ethics committee N°005.

## Author Contributions

VD, CB, KD, M-CN, MS, NA, AM, and EG performed the experiments. CA participated to *in vivo* experiments. TA and SJ synthetized all forms of recFH and recFH fragments. VD, SJ, FB-C, FM, TA, and CB conceptualized experiments. VD, FB-C, CB, TA, FM, SJ, YS, LK, and PL interpreted data. VD, SJ, FM, and FB-C wrote the manuscript. VD was responsible for research supervision. FB-C was an RPIB coordinator for this project funding.

### Conflict of Interest

The authors declare that the research was conducted in the absence of any commercial or financial relationships that could be construed as a potential conflict of interest.

## Supplementary Material

Supplementary Data 1 Production and *in vitro* characterization of the full-length recFH and fragments. **(A)** SDS-PAGE analysis of FH purified from human plasma (plFH) and from culture supernatant of PER.C6 (recFH1-20). **(B)** Main characteristics of recFH and fragments (sequence, molecular weigth) and *in vitro* activities (decay accelerating C3-convertase and protecting sheep erythrocytes lysis test) were used to determine FH capacity to inhibit AP activity in fluid phase and on cell surface. EC_50_ refers to the concentration of recFH or its fragments necessary to accelerate the dissociation of C3 convertase.

Supplementary Data 2 Detection of recFH in RPE/choroid/sclera complex of native rat. **(A,B)** On western blot plFH, recFH1-20 or recFH fragments levels were observed in the RPE/choroid/sclera complex of native rats after 1, 24, and 72 h post-IVT injection. A specific human anti-FH antibody which does not cross-react with endogenous rat FH was used. Injection of plFH, recFH, or recFH fragments were realized in both eye of 4 animals per experimental group. Data represented a representative experiment (from two independent experiments). CNV kinetic analysis by western blotting **(C)** of endogenous FH (rat-FH) production and **(D)** of IVT injected recFH1-20 (0.6 μM). Semi-quantifications of FH (rat and human recombinant)/actin levels were done for each time point of CNV process. Ten impacts per eye of each 4 animals experimental group were realized and experiments were done 3 times. Data were analyzed and compared using Mann–Whitney *U*-test and differences are considered statistically significant with ^**^*P* < 0.01; ^***^*P* < 0.001.

Supplementary Data 3 Test of the anti-FH antibodies specificity. **(A)** Immunostaining of endogenous FH [anti-FH (Rat)] and of IVT injected recFH1-20 [6 μM, anti-FH (Human)] on CNV laser sections were studied at day 14 post-laser. IVT injection (recFH or PBS as control) were done at day 4 post-laser in each rat eye of 4 animals per experimental group. Five laser impacts per eye were realized in each group. Experiments were done 3 times. Scale Bar: 50 μm. **(B)** As a control, the primary antibody was omitted and no staining was observed in any control. Experiments were done 3 times. Scale Bar: 50 μm.

Supplementary Data 4 FH up-regulates *tsp-1* gene expression in rat CNV model. Eye of rat CNV model were IVT injected with recFH (1–20 or 7–20, 0.6 μM) at day 4 post-laser and *tsp-1* gene expression was analyzed by Q-PCR experiments at day 7 post-laser. Ten laser impacts per eye were realized in each 4 animals experimental group and this experiment was repeated 3 times. Data were analyzed and compared using Mann–Whitney *U*-test and differences are considered statistically significant with ^*^*P* < 0.05; ^**^*P* < 0.01.

Supplementary Data 5 Analysis of antibodies specificity. **(A)** As a control, the primary antibody was omitted and no staining was observed in any lesion spot induced by laser in rat CNV model. Scale Bar: 50 μm. **(B)** Analysis of the effect of mouse IgG IVT injection in eye of rat CNV model. FITC-ISB4 on RPE/choroid/sclera flat mounting laser spot staining was realized 14 days after laser for each experimental group. For treatment, at day 4 post-laser, each 4 animals per experimental group received an IVT injection per eye of recFH1-20 (0.6 μM) or recFH7-20 (0.6 μM) and 10 min later an IVT injection of mouse IgG (6 μM). For control, each eye of animals (*n* = 4) were IVT injected first with PBS and 10 min later with mouse IgG (6 μM). Five impacts per eye in each 4 animals experimental group were realized. Experiments were performed 3 times. ISB4-stained CNV areas were expressed as mean ± SEM of average CNV size per animal. Linear mixed model was used for statistical analyses ^***^*p* < 0.001. Scale Bar: 100 μm.

## Abbreviations

AMD: Age-related Macular Degeneration
nAMD/wet AMD: neo-vascular AMD
AP: Alternative Pathway of the complement system
C3: Complement protein 3
CCP: Complement Control Protein
CNV: Choroidal NeoVascularization
CR3: Complement receptor 3
FH: Factor H
FHL-1: FH Like protein 1
recFH: recombinant Factor H
plFH: plasma Factor H
GAG: GlycosAminoGlycans
IVT: Intravitreous Injection
MAC/C5b-9: Membrane attack Complex
RPE: Retinal Pigment Epithelium
TSP-1: Thrombospondin-1
VEGF: Vascular Endothelial Growth Factor.
