# Supplementary figures and images for: Mechanisms of FH Protection Against Neovascular AMD
Source: Front Immunol. 2020 Apr 3;11:443. doi: 10.3389/fimmu.2020.00443 (PMC7146894; doi:10.3389/fimmu.2020.00443)

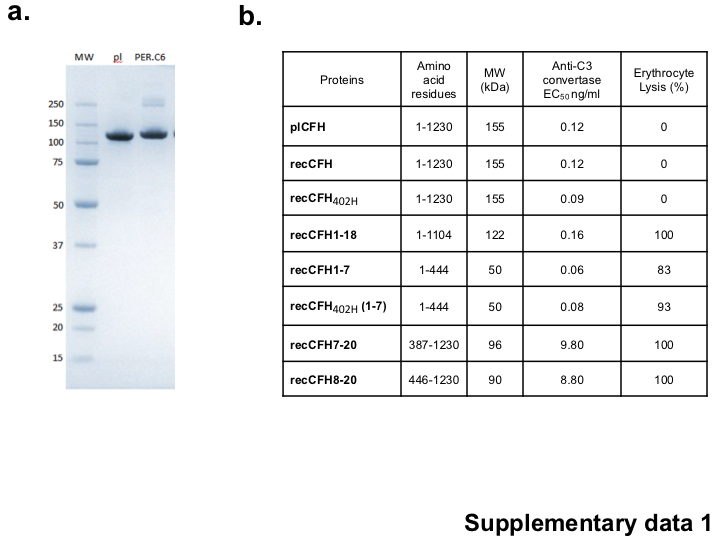

Supplement: Supplementary Data 1 — Production and in vitro characterization of the full-length recFH and fragments. (A) SDS-PAGE analysis of FH purified from human plasma (plFH) and from culture supernatant of PER.C6 (recFH1-20). (B) Main characteristics of recFH and fragments (sequence, molecular weigth) and in vitro activities (decay accelerating C3-convertase and protecting sheep erythrocytes lysis test) were used to determine FH capacity to inhibit AP activity in fluid phase and on cell surface. EC50 refers to the concentration of recFH or its fragments necessary to accelerate the dissociation of C3 convertase. [file Image_1.TIFF]

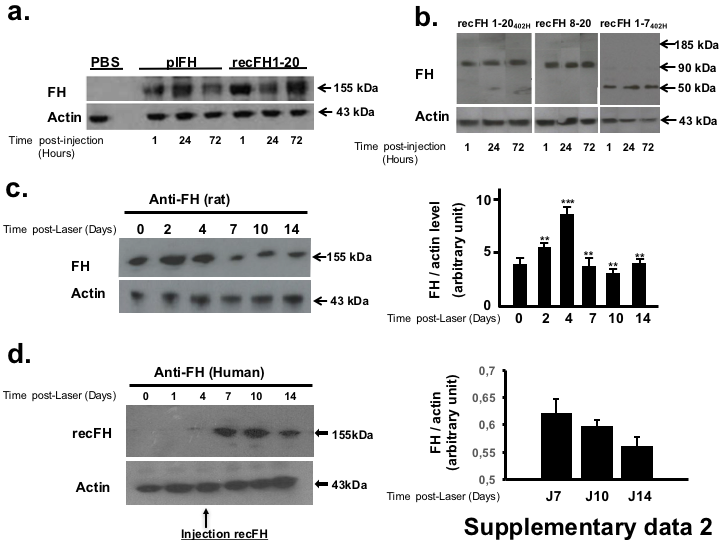

Supplement: Supplementary Data 2 — Detection of recFH in RPE/choroid/sclera complex of native rat. (A,B) On western blot plFH, recFH1-20 or recFH fragments levels were observed in the RPE/choroid/sclera complex of native rats after 1, 24, and 72 h post-IVT injection. A specific human anti-FH antibody which does not cross-react with endogenous rat FH was used. Injection of plFH, recFH, or recFH fragments were realized in both eye of 4 animals per experimental group. Data represented a representative experiment (from two independent experiments). CNV kinetic analysis by western blotting (C) of endogenous FH (rat-FH) production and (D) of IVT injected recFH1-20 (0.6 μM). Semi-quantifications of FH (rat and human recombinant)/actin levels were done for each time point of CNV process. Ten impacts per eye of each 4 animals experimental group were realized and experiments were done 3 times. Data were analyzed and compared using Mann–Whitney U-test and differences are considered statistically significant with **P < 0.01; ***P < 0.001. [file Image_2.TIFF]

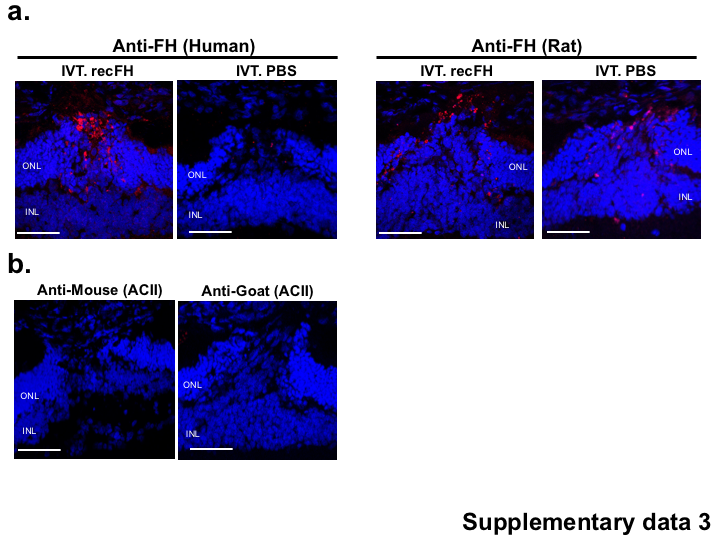

Supplement: Supplementary Data 3 — Test of the anti-FH antibodies specificity. (A) Immunostaining of endogenous FH [anti-FH (Rat)] and of IVT injected recFH1-20 [6 μM, anti-FH (Human)] on CNV laser sections were studied at day 14 post-laser. IVT injection (recFH or PBS as control) were done at day 4 post-laser in each rat eye of 4 animals per experimental group. Five laser impacts per eye were realized in each group. Experiments were done 3 times. Scale Bar: 50 μm. (B) As a control, the primary antibody was omitted and no staining was observed in any control. Experiments were done 3 times. Scale Bar: 50 μm. [file Image_3.TIFF]

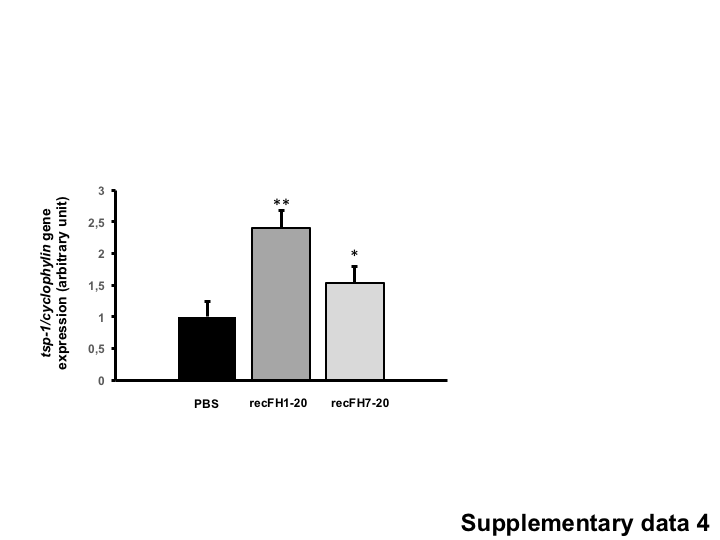

Supplement: Supplementary Data 4 — FH up-regulates tsp-1 gene expression in rat CNV model. Eye of rat CNV model were IVT injected with recFH (1–20 or 7–20, 0.6 μM) at day 4 post-laser and tsp-1 gene expression was analyzed by Q-PCR experiments at day 7 post-laser. Ten laser impacts per eye were realized in each 4 animals experimental group and this experiment was repeated 3 times. Data were analyzed and compared using Mann–Whitney U-test and differences are considered statistically significant with *P < 0.05; **P < 0.01. [file Image_4.TIFF]

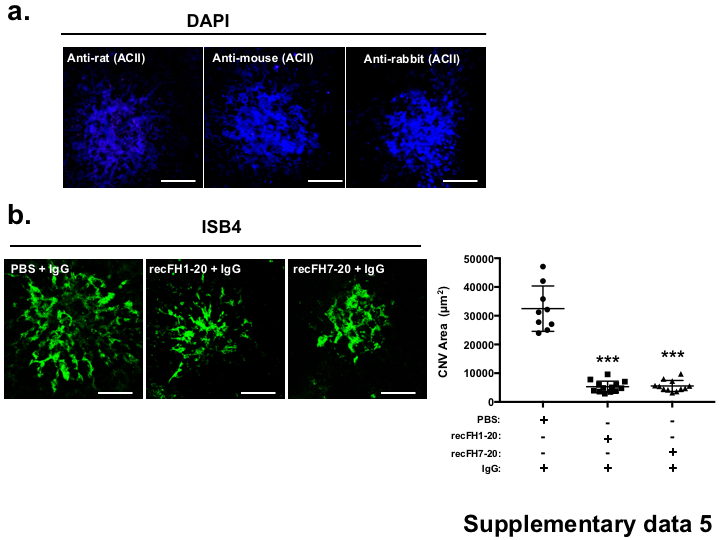

Supplement: Supplementary Data 5 — Analysis of antibodies specificity. (A) As a control, the primary antibody was omitted and no staining was observed in any lesion spot induced by laser in rat CNV model. Scale Bar: 50 μm. (B) Analysis of the effect of mouse IgG IVT injection in eye of rat CNV model. FITC-ISB4 on RPE/choroid/sclera flat mounting laser spot staining was realized 14 days after laser for each experimental group. For treatment, at day 4 post-laser, each 4 animals per experimental group received an IVT injection per eye of recFH1-20 (0.6 μM) or recFH7-20 (0.6 μM) and 10 min later an IVT injection of mouse IgG (6 μM). For control, each eye of animals (n = 4) were IVT injected first with PBS and 10 min later with mouse IgG (6 μM). Five impacts per eye in each 4 animals experimental group were realized. Experiments were performed 3 times. ISB4-stained CNV areas were expressed as mean ± SEM of average CNV size per animal. Linear mixed model was used for statistical analyses ***p < 0.001. Scale Bar: 100 μm. [file Image_5.TIFF]
